# Altered platelet and coagulation function in moderate-to-severe COVID-19

**DOI:** 10.1038/s41598-021-95397-6

**Published:** 2021-08-11

**Authors:** Rustem I. Litvinov, Natalia G. Evtugina, Alina D. Peshkova, Svetlana I. Safiullina, Izabella A. Andrianova, Alina I. Khabirova, Chandrasekaran Nagaswami, Rafael R. Khismatullin, Svetlana S. Sannikova, John W. Weisel

**Affiliations:** 1 Department of Cell and Developmental Biology, University of Pennsylvania School of Medicine, Philadelphia, PA USA; 2 Institute of Fundamental Medicine and Biology, Kazan Federal University, Kazan, Russian Federation; 3 Medical Center “Aibolit”, Kazan, Russian Federation; 4 City Hospital, No.16, Kazan, Russian Federation; 5 Department of Cell and Developmental Biology, University of Pennsylvania Perelman School of Medicine, 421 Curie Blvd., BRB II/III, Room 1153, Philadelphia, PA 19104 USA

**Keywords:** Infection, Prognostic markers

## Abstract

To reveal if coagulopathies relate to the course of COVID-19, we examined 255 patients with moderate and severe COVID-19, receiving anticoagulants and immunosuppressive drugs. Coagulopathy manifested predominantly as hypercoagulability that correlated directly with systemic inflammation, disease severity, comorbidities, and mortality risk. The prolonged clotting tests in about ¼ of cases were associated with high levels of C-reactive protein and antiphospholipid antibodies, which impeded coagulation in vitro. Contraction of blood clots was hindered in about ½ of patients, especially in severe and fatal cases, and correlated directly with prothrombotic parameters. A decrease in platelet contractility was due to moderate thrombocytopenia in combination with platelet dysfunction. Clots with impaired contraction were porous, had a low content of compressed polyhedral erythrocytes (polyhedrocytes) and an even distribution of fibrin, suggesting that the uncompacted intravital clots are more obstructive but patients could also be prone to bleeding. The absence of consumption coagulopathy suggests the predominance of local and/or regional microthrombosis rather than disseminated intravascular coagulation. The results obtained (i) confirm the importance of hemostatic disorders in COVID-19 and their relation to systemic inflammation; (ii) justify monitoring of hemostasis, including the kinetics of blood clot contraction; (iii) substantiate the active prophylaxis of thrombotic complications in COVID-19.

## Introduction

The infectious disease caused by the SARS-CoV-2 virus has been named COVID-19. Despite numerous studies, the results of COVID-19 treatment remain mediocre, mainly due to the lack of detailed information on the pathogenesis of the disease, which is different from other known infections in many respects. The SARS-CoV-2 virus directly or indirectly affects various organs and systems, including hemostasis^1^. Macro- and microthrombosis in COVID-19 dictate thromboprophylaxis with low molecular weight heparin (LMWH)^2–4^. Anticoagulation significantly reduced the mortality rates^5^, thus confirming the importance of thrombosis in the pathophysiology of COVID-19. In addition to the pathogenic and clinical importance of thrombotic complications, laboratory hemostatic tests have become valuable criteria for prognosis and treatment effectiveness^6^ and high levels of fibrinogen and D-dimer serve as an indication for aggressive anticoagulation in COVID-19^7^.

Despite the proven role of coagulopathies in the clinical course and outcomes of COVID-19^8–12^, some aspects of the pathogenesis of hemostatic disorders in COVID-19 remain understudied. It is not clear if there is a causal relation of hemostatic disorders with local and systemic inflammation and what is the role of inflammatory mediators in the initiation of hypercoagulability and sustaining a high thrombotic potential. A direct correlation between the hypercoagulability and the severity of the disease is not obvious. It is unclear whether there is a link between the high thrombotic potential and deaths, especially during heparin therapy. It is important to elucidate the relative utility of hemostatic laboratory tests. Clarifying these and other questions was the objective of this study.

Here, using a unique set of complementary techniques, we have shown that the hemostatic disorders in COVID-19 largely determine the course and outcomes of the disease. Multiple laboratory signs of hypercoagulability persist in the moderate and severe forms of COVID-19, notwithstanding administration of LMWH and anti-inflammatory drugs. The hemostatic disorders are strongly related to the systemic inflammation that not only triggers the blood clotting system, but also interferes with the hemostatic chronometric tests via direct anticoagulant effects of C-reactive protein (CRP) and antiphospholipid antibodies. The pro-thrombotic state in COVID-19 comprises an important pathophysiological mechanism, underlying a risk of imminent or ongoing thrombosis that may expand to disseminated intravascular coagulation (DIC) in critical conditions. Therefore, patients with moderate and severe COVID-19 require continuous laboratory monitoring of hemostasis and personalized use of anticoagulants to prevent or cure overt and latent thrombotic complications.

## Results

### Thrombodynamics analysis in patients with COVID-19

**Figure 1:**
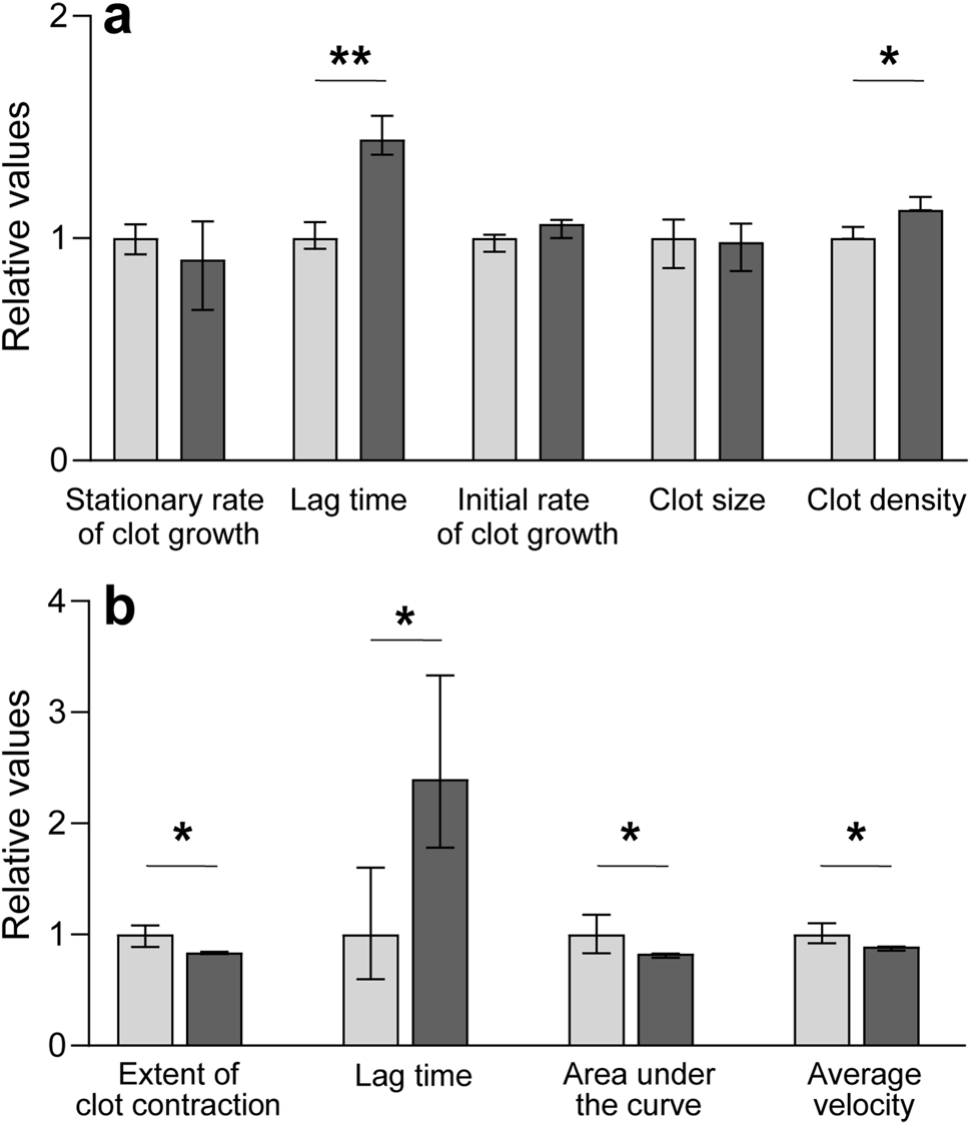
Parameters of thrombodynamics (**a**) and kinetics of blood clot contraction (**b**) in COVID-19 patients normalized by the values for healthy subjects (the median taken as 1). Grey bars—healthy subjects (n = 20 for **a** and 40 for **b**﻿); dark bars—patients with COVID-19 (n = 200 for **a** and 215 for **b**). The results are presented as median and interquartile range (25th and 75th percentiles). *p < 0.05; ** p < 0.01; Mann–Whitney U-test.

The thrombodynamics assay, which tracks the spatial growth of a plasma clot (see Methods), revealed that patients with COVID-19 had a significant increase in the clot optical density (Fig. 1a, Table 1a), which was due to hyperfibrinogenemia. At the fibrinogen levels < 4 g/l, the median density was 24,400 a.u. (IQR 21,300; 27,500), while at the levels > 4 g/l the clot density was 30,500 a.u. (IQR 27,700; 32,600; p < 0.001), indicating a direct dependence of the clot optical density on the fibrin(ogen) content.

**Table 1:** Parameters of thrombodynamics in the COVID-19 patients.

Comparison groups and subgroups	Parameters of thrombodynamics				
	Stationary rate of clot growth, μm/min	Lag time, min	Initial rate of clot growth, μm/min	Clot size, μm	Clot density, a.u
**(a) Patients with COVID-19 and healthy donors**					
Patients with COVID-19 (n = 200)	25 (18; 32)	1.3 (1.1; 1.6)***	54 (50; 60)	1103 (901; 1253)	29,100 (24,900; 32,800)**
Healthy subjects (n = 20)	28 (26; 30)	0.9(0.8; 1.0)	51 (50; 55)	1122 (1057; 1175)	25,800 (21,000; 28,300)
**(b) Dependence on the outcome of the disease**					
Favorable (n = 184)	25.3 (18.2; 32.1)*	1.3 (1.1; 1.5)	54.7 (49.9; 59.8)*	1108 (917; 1279)**	29,000 (24,800; 31,600)*
Fatal (n = 16)	20.1 (15.4; 25.2)	1.3 (1.1; 1.7)	50.2 (45.1; 53.2)	910 (871; 1055)	32,100 (28,300; 34,800)
**(c) Dependence on the severity of the disease**					
Moderate severity (n = 149)	25.5 (18.0; 32.2)	1.3 (1.1; 1.5)	54.9 (49.9; 59.7)	1130 (909; 1288)**	29,000 (25,200; 31,500)
Severe (n = 51)	25.5 (18.0; 32.2)	1.3 (1.2; 1.6)	52.2 (49.2; 57.8)	985 (881; 1118)	29,500 (24,100; 32,800)
**(d) Dependence on the presence of concomitant hematologic neoplasms**					
Neoplasms (n = 8)	32.3* (24.9; 35.8)	1.1 (0.9; 1.3)	66.4 (55.8; 68.4)**	1364 (1109; 1449)**	26,100 (24,400; 29,400)
No neoplasms (n = 207)	24.9 (17.0; 31.4)	1.3 (1.1; 1.6)	53.9 (49.3; 59.6)	1098 (894; 1238)	29,100 (25,000; 31,900)
**(e) Dependence on the level of C-reactive protein in blood serum**					
< 10 mg/l (n = 23)	32.1 (21.2; 36.5)*	1.1 (1.0; 1.2)**	61.3 (50.9; 66.5)**	1276 (930; 1408)*	27,000 (23,300; 29,700)**
> 10 mg/l (n = 114)	23.9 (17.6; 28.7)	1.3 (1.1; 1.5)	54.0 (49.8; 58.9)	1082 (917; 1183)	30,400 (26,400; 32,600)

Results are presented as median and interquartile range (25th and 75th percentiles).

*p < 0.05, **p < 0.01, ***p < 0.001; Mann–Whitney U-test.

In 15% of cases, formation of “spontaneous” clots was observed in the bulk plasma that was not in contact with the tissue factor-coated insert, which is a sign of enhanced thrombin generation. Paradoxically, despite the “spontaneous” plasma clots, there were no significant differences in the kinetic parameters of the thrombodynamics assay, such as the initial and stationary clot growth rates. Moreover, there was a significantly longer median lag-time (Fig. 1a, Table 1a), which paralleled the prolonged prothrombin time and an increase in the INR (Table 2).

**Table 2:** Hemostatic parameters in COVID-19 patients and healthy subjects.

Parameters	COVID-19 patients (n = 215)	Healthy subjects (n = 40)
TEG parameters		
*R*, min	5.8 (4.8; 6.8)***	4 (3.5; 4.3)
*K*, min	0.9 (0.8; 1.2)***	2.2 (1.3; 4.4)
Angle αº	75.6 (70.2; 78.2)	74.3 (72.1; 76.2)
*MA*, mm	38.6 (33.5; 44.5)***	22.7 (20.4; 25.9)
Fibrinogen, g/l	5.1 (3.7; 5.9)*	4.0 (3.4; 4.5)
D-dimer, ng/ml	709 (431; 1545)***	223 (149; 406)
Prothrombin time, sec	12.0 (11.0; 13.5)***	11.0 (10.6; 11.3)
INR	1.02 (0.99; 1.08)**	0.97 (0.94; 1.04)
Soluble fibrin complexes (positive ethanol gelation test)	20%	0
Platelet count, × 10^9^/l	225 (158; 292)*	247 (220; 300)

Results are presented as median and interquartile range (25th and 75th percentiles).

*p < 0.01, **p < 0.001, ***p < 0.0001; Mann–Whitney U-test.

The thrombodynamic parameters depended on the severity of the disease. In patients with a lethal outcome, a significant delay in clot formation was observed with a decrease in the rates of clot growth and a corresponding decrease in clot size (Table 1b, Fig. S1a). Accordingly, in the severe course of the disease, the median clot size was decreased significantly compared with the moderate-to-severe course (Table 1c). These results indicate that in severe cases of COVID-19, the thrombodynamic parameters shift towards hypocoagulability. This transformation was not due to the fact that critically ill patients received higher doses of LMWH. It is more likely that the hypocoagulability is associated with high levels of CRP and antiphospholipid antibodies, which are known to decelerate clotting in vitro^13,14^.

The results of the thrombodynamics assay were affected by concomitant diseases. Thus, in COVID-19 patients with pre-existing neoplasms of hematopoietic or lymphoid tissues, the clot growth rates and clot size were increased significantly (Table 1d), indicating pronounced hypercoagulability compared to the isolated COVID-19 cases. Most likely, it was due to the summation of various pathogenetic factors that activate the clotting cascade. Notably, patients with hematologic neoplasms were not on heparin because of severe thrombocytopenia, while patients with isolated COVID-19 received LMWH.

### Thrombodynamics analysis and inflammation in COVID-19

Correlation analysis of the thrombodynamics parameters and laboratory signs of inflammation revealed important connections (Table S1). A positive correlation was found between the clot optical density and CRP and erythrocyte sedimentation rate (ESR); the clot size correlated positively with the blood monocyte counts. A direct correlation was found between interleukin-8 and the initial velocity of clot formation and clot size. The abnormally high levels of interleukin-8 (> 10 pg/ml) were associated with higher initial rates of clot growth with a median of 54.4 μm/min (IQR 51.3; 58.8) versus 49.2 μm/min (IQR 43.5; 52.5; p < 0.005) when interleukin-8 was normal (< 10 pg/ml), suggesting inflammation-induced hypercoagulability with a risk of immunothrombosis as a result of generalized activation of IL-8-producing innate immune cells.

An increase in CRP > 10 mg/l was paradoxically associated with a decrease in the clot growth rates and intermediate clot size as well as with prolonged lag time (Table 1e). A combination of hypocoagulability with the high levels of CRP in the blood agrees with the ability of CRP to decelerate blood clotting in vitro by blocking procoagulant phospholipids^15,16^. Concomitantly with increased levels of CRP > 10 mg/l, the clot optical density increased, reflecting hyperproduction of fibrinogen, which is, like CRP, an acute phase protein, and both are upregulated by interleukin-6^17^. A related finding was that the initial rate of clot growth was significantly smaller in patients with abnormally elevated levels of anti-cardiolipin antibodies (Fig. S2) and the two parameters correlated inversely (r = -0.39; p = 0.0081). Thus, there is close relationship between coagulopathies and inflammation in COVID-19.

To reveal potential effects of LMWH on the parameters measured, the COVID-19 patients were segregated into the subgroups receiving standard prophylactic, high prophylactic, and therapeutic doses of LMWH (Table S2). With the exception of the lag time and clot size in the thrombodynamics assay that were sensitive to increased doses of LMWH, there was no significant difference in the hemostatic parameters tested between the subgroups of patients receiving various doses of LMWH. Therefore, administration LMWH, either at a prophylactic or therapeutic dose, did not fully prevent coagulopathies in severe COVID-19, indicating strong and persistent procoagulant and prothrombotic potential in systemic inflammation induced by SARS-CoV-2.

### Clot contraction kinetics in clots made from the blood of patients with COVID-19

The formation and shrinkage of blood clots were induced by exogenous thrombin followed by optical tracking of the clot size and measuring the kinetics of clot contraction. In the COVID-19 patients, a pronounced, significant inhibition of all stages of the blood clot contraction process was found (Figs. 1b and S1b, Table 3a). To exclude or minimize potential effects of the residual LMWH activity, the patients’ blood samples with a reduced extent of contraction were re-tested after pre-treatment with protamine sulfate. There were 11 (9.7%) out of 113 blood samples in which the extent of contraction was partially normalized after neutralization of heparin. These samples were excluded from data analysis to ensure that the measurements were not affected by LMWH.

**Table 3:** Parameters of blood clot contraction in the COVID-19 patients.

Comparison groups and subgroups	Parameters of blood clot contraction			
	Extent of clot contraction, %	Lag time, sec	Area under the curve, a.u	Average velocity, %/s × 10^–3^
**(a) Patients with COVID-19 and healthy donors**				
Patients with COVID-19 (n = 215)	41 (37; 43)***	180 (150; 213)***	315 (261; 354)***	34 (31; 36)***
Healthy subjects (n = 40)	48 (43; 52)	75 (45; 120)	380 (316; 447)	38 (35; 42)
**(b) Dependence on the severity of the disease**				
Moderate severity (n = 149)	41 (38; 44)*	180 (150; 195)**	322 (280; 358)**	34 (32; 36)*
Severe (n = 51)	39 (33; 43)	210 (165; 255)	298 (220; 337)	32 (27; 35)
**(c) Dependence on the outcome of the disease**				
Favorable (n = 184)	41 (38; 44)*	180 (150; 210)***	317 (271; 357)**	34 (31; 36)**
Fatal (n = 16)	35 (27; 42)	255 (195; 349)	238 (156; 320)	30 (22; 35)
**(d) Dependence on the presence of concomitant hematologic neoplasms**				
Neoplasms (n = 8)	28 (17; 42)*	218 (150; 251)	200 (117;304)**	23 (14; 34)
No neoplasms (n = 207)	41 (37; 44)	180 (150; 210)	316 (270; 357)	34 (31; 36)
**(e) Dependence on the presence of concomitant coronary heart disease**				
Coronary heart disease (n = 41)	39 (33; 41)**	195 (165; 240)*	298 (227; 335)*	32 (29; 35)*
No coronary heart disease (n = 159)	41 (38; 44)	180 (150; 210)	324 (276; 361)	34 (32; 36)
**(f) Dependence on the presence of concomitant ischemic stroke or transient ischemic attacks**				
Ischemic stroke or transient ischemic attacks (n = 23)	36 (27; 43)*	225 (165; 285)**	272 (152; 318)*	30 (22; 36)*
No ischemic stroke or transient ischemic attacks (n = 162)	41 (38; 44)	180 (150; 210)	323 (279; 358)	34 (31; 36)
**(g) Dependence on the gender**				
Women (n = 115)	42 (39; 45)***	180 (150; 225)	323 (291; 371)**	34 (32; 37)***
Men (n = 100)	39 (35; 42)	180 (150; 210)	306 (253; 341)	33 (29; 35)

Results are presented as median and interquartile range (25th and 75th percentiles).

*p < 0.05, **p < 0.01, ***p < 0.001; Mann–Whitney U-test.

The impaired clot contraction was associated directly with disease severity and poor outcomes. In patients with severe disease, blood clot contraction was suppressed significantly compared to patients with moderate disease manifestations (Table 3b). In addition, in the critical category of patients on mechanical ventilation, the lag time of contraction was slowed down significantly (median 240 s, IQR 195; 465) compared with patients who were not on ventilation (180 s, IQR 150; 210; p < 0.01). Accordingly, in patients with lethal outcomes, clot contraction was significantly impaired compared to those who survived (Table 3c). The strong relation between the clot contraction kinetics and the severity and outcomes of COVID-19 suggests an important pathophysiological role of impaired clot contraction and the prognostic value of the clot contraction assay.

The parameters of blood clot contraction depended on comorbidities. In patients with COVID-19 and concomitant hematologic neoplasms, clot contraction was significantly reduced and delayed (Table 3d). Similar suppression of clot contraction was observed when COVID-19 was associated with ischemic heart disease (Table 3e) and a history of cerebrovascular accidents (Table 3f). These results confirm our previous studies that clot contraction is impaired in venous thromboembolism^18,19^ and acute ischemic stroke^20^. Notably, in male patients with COVID-19, clot contraction was significantly weakened compared to women (Table 3g).

### Relationship of clot contraction to blood composition in COVID-19

**Figure 2:**
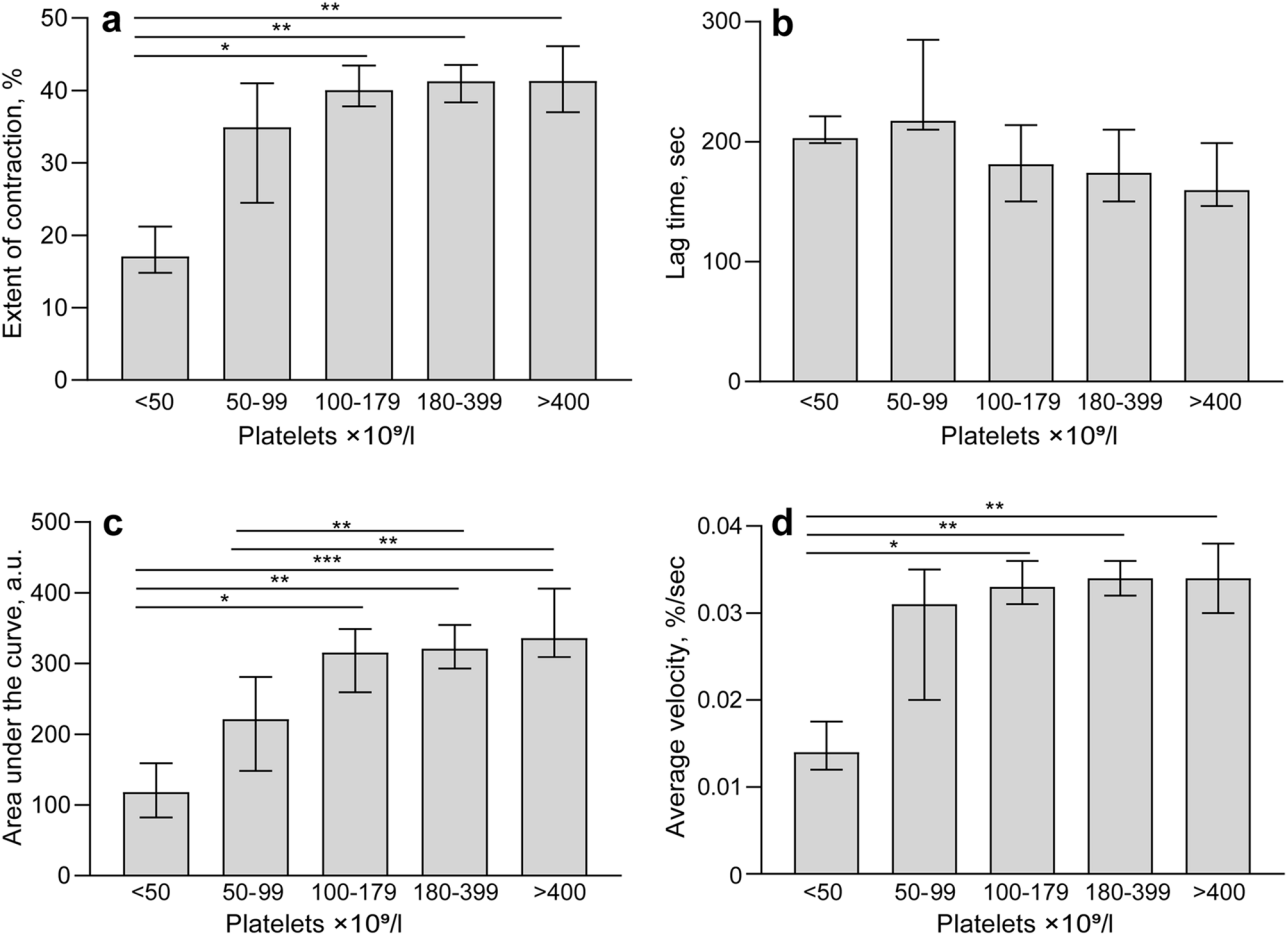
Parameters of blood clot contraction as a function of platelet counts in the blood of COVID-19 patients. (**a**) Extent of clot contraction; (**b**) lag time; (**c**) area under the kinetic curve (equivalent to work performed by platelets); (**d**) average contraction velocity. The results are presented as median and interquartile range (25th and 75th percentiles). *p < 0.05, **p < 0.01, ***p < 0.001; Kruskal–Wallis test with Tukey's multiple comparisons post hoc test.

The rate and extent of clot contraction depend on the quantity and quality of platelets, as well as on pathological alterations of blood composition^18^; therefore, the parameters of clot contraction were compared with hematologic tests (Table S4). There was a strong dependence of blood clot contraction (except the lag time) on the platelet counts (Fig. 2a). A decrease in clot contraction was observed at moderate thrombocytopenia of (100–150) × 10^9^/l; at a platelet count of 50 × 10^9^/l and lower there was a progressive weakening and almost complete suppression of clot contraction. These data confirm the key role of platelets in clot contraction and the important role of thrombocytopenia in COVID-19. Expectedly, platelet counts and thrombocrit values correlated positively with the extent of contraction and area under the kinetic curves (Table S4). The lag time related inversely to the platelet count and correlated positively with prothrombin time, unlike the area under the kinetic curves that correlated negatively with prothrombin time.

Notably, the extent of clot contraction correlated negatively with the mean volume of erythrocytes and the mean hemoglobin content. The velocity of clot contraction correlated negatively with the erythrocyte volume and the hemoglobin content (Table S4). In addition to the correlations described, anemia at a hemoglobin level < 110 g/l was associated with a prolonged lag time with a median of 203 s (IQR 165; 251), whereas at normal hemoglobin levels > 110 g/l, the lag time shortened to 180 s (IQR 150; 210; p < 0.01). The relationship of the clot contraction kinetics to various hematologic and hemostatic parameters indicates that the determinants of clot contraction are not limited to platelet count and include variations in erythrocyte count and hemoglobin content. In addition, there was a strong association between clot contraction and the levels of D-dimer. Specifically, among patients with reduced extent of contraction below normal (< 41%, see Methods), those with an increased level of D-dimer (> 1000 ng/ml) prevailed over the patients with normal contraction ≥ 41% (p < 0.05; χ^2^-test), which supports the association of impaired contraction of blood clots with fibrin deposition reflected by a high D-dimer.

### Structure and composition of clots formed from the blood of patients with COVID-19

The main structural elements of blood clots are fibrin fibers, erythrocytes, and activated platelets^21^. In the contracted blood clots, erythrocytes can be deformed and segregated into four morphological types: (1) biconcave (original, undeformed); (2) partially deformed, but close to the original ("intermediate biconcave"); (3) more deformed, acquiring the features of polyhedra ("intermediate polyhedral") and (4) maximally deformed to the stage of polyhedra ("polyhedrocytes")^22^.

**Figure 3:**
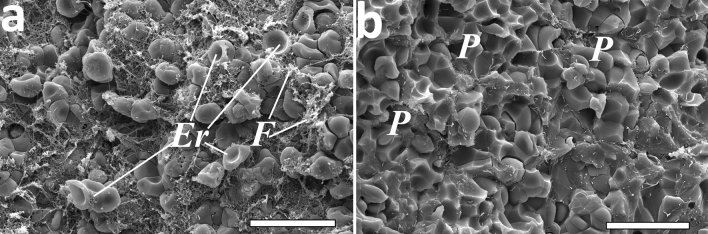
Representative scanning electron micrographs of in vitro blood clots from COVID-19 patients with a low (13%) (**a**) and high (49%) (**b**) extent of clot contraction. *Er*—partially deformed erythrocytes, *F*—fibrin, *P*—fully deformed polyhedral erythrocytes (polyhedrocytes). Magnification bars: 15 µm.

The composition of clots formed from the blood of COVID-19 patients varied greatly, depending on the clots’ ability to contract. The differences could be clearly seen by comparing two clots with the lowest (13%) and highest (49%) extents of contraction, both shown in Fig. 3. When contraction was weak (Fig. 3a), the clot was porous with a high content of fibrin and a lack of deformed cells, while maintaining uncompressed biconcave erythrocytes. At a high degree of compression (Fig. 3b), the clot became dense, non-porous, with a high content of deformed polyhedrocytes, but without biconcave erythrocytes. In addition, clot contraction caused redistribution of fibrin, which accumulated at the outside of the clot.

These structural differences could be generalized after quantitative analysis of the composition of clots made from the blood of patients with a normal (≥ 41%, see “Methods”) and reduced (< 41%) extent of blood clot contraction, the latter being found in 53% of the patients with COVID-19 examined (Table S5). In the normally contracted clots, erythrocytes undergoing compressive deformation predominated, fibrin accumulated on the periphery of the clot, and the porosity was reduced. On the contrary, in clots with impaired contraction, the proportion of deformed (multifaceted) erythrocytes was low, fibrin was randomly distributed throughout the clot, and the porosity was relatively high. The variations revealed in the morphology of blood clots provide a structural basis for the pathophysiological consequences of the impaired contraction of blood clots in patients with COVID-19.

### Platelet function and morphology in COVID-19

COVID-19 patients typically had moderate thrombocytopenia (Table 2) without hemorrhage, because platelet counts rarely fell below 100 × 10^9^/l. To assess platelet quality, we determined the expression of P-selectin, active integrin αIIbβ3, and phosphatidylserine (flow cytometry), secretion of soluble P-selectin (sP-selectin) (ELISA), and platelet morphology (scanning electron microscopy).

Flow cytometry (Table S6) showed that COVID-19 patients had a twofold increased fraction of “spontaneously” activated platelets expressing phosphatidylserine and P-selectin on their surface. After 3 min of TRAP-induced activation, P-selectin was expressed on about 85% of platelets in COVID-19 patients, which was significantly higher compared to the control. This difference in the fraction of P-selectin-expressing platelets vanished after a 10-min treatment with TRAP-6. Moreover, after 10-min of incubation with TRAP-6, the fraction of platelets expressing activated integrin αIIbβ3 was significantly smaller in COVID-19 patients compared to healthy controls. These results suggest that in the blood of COVID-19 patients, there is mild background activation of circulating platelets associated with concurrent partial platelet refractoriness.

Expression of P-selectin on the platelet surface is coupled with secretion of the soluble form of P-selectin (sP-selectin) into the blood, which can be used as a marker of platelet activation^23^. COVID-19 patients had higher levels of sP-selectin (median 81 ng/ml; IQR 55; 104) compared with healthy subjects (55 ng/ml; IQR 31; 70; p < 0.001). Importantly, the level of sP-selectin in serum correlated positively with the blood counts of inflammatory cells, namely neutrophils (r = 0.53; p < 0.0001), lymphocytes (r = 0.29; p < 0.05), and monocytes (r = 0.25; p < 0.05). Furthermore, the elevated levels of sP-selectin (82.5 ng/ml; IQR 64.6; 111.6) were associated with abnormally high levels of interleukin-8 (> 35 pg/ml), while in patients who had lower levels of interleukin-8 (< 35 pg/ml) the levels of sP-selectin were significantly lower (58.2; IQR 50.2; 87.5; p < 0.05). Another observation supporting the importance of inflammatory cytokines in COVID-19 was that patients with interleukin-8 > 35 pg/ml had significantly lower levels of blood oxygen saturation (median 94%; IQR 92; 96), compared with patients who had interleukin-8 < 35 pg/ml (median 96%; IQR 93; 97; p < 0.05).

**Figure 4:**
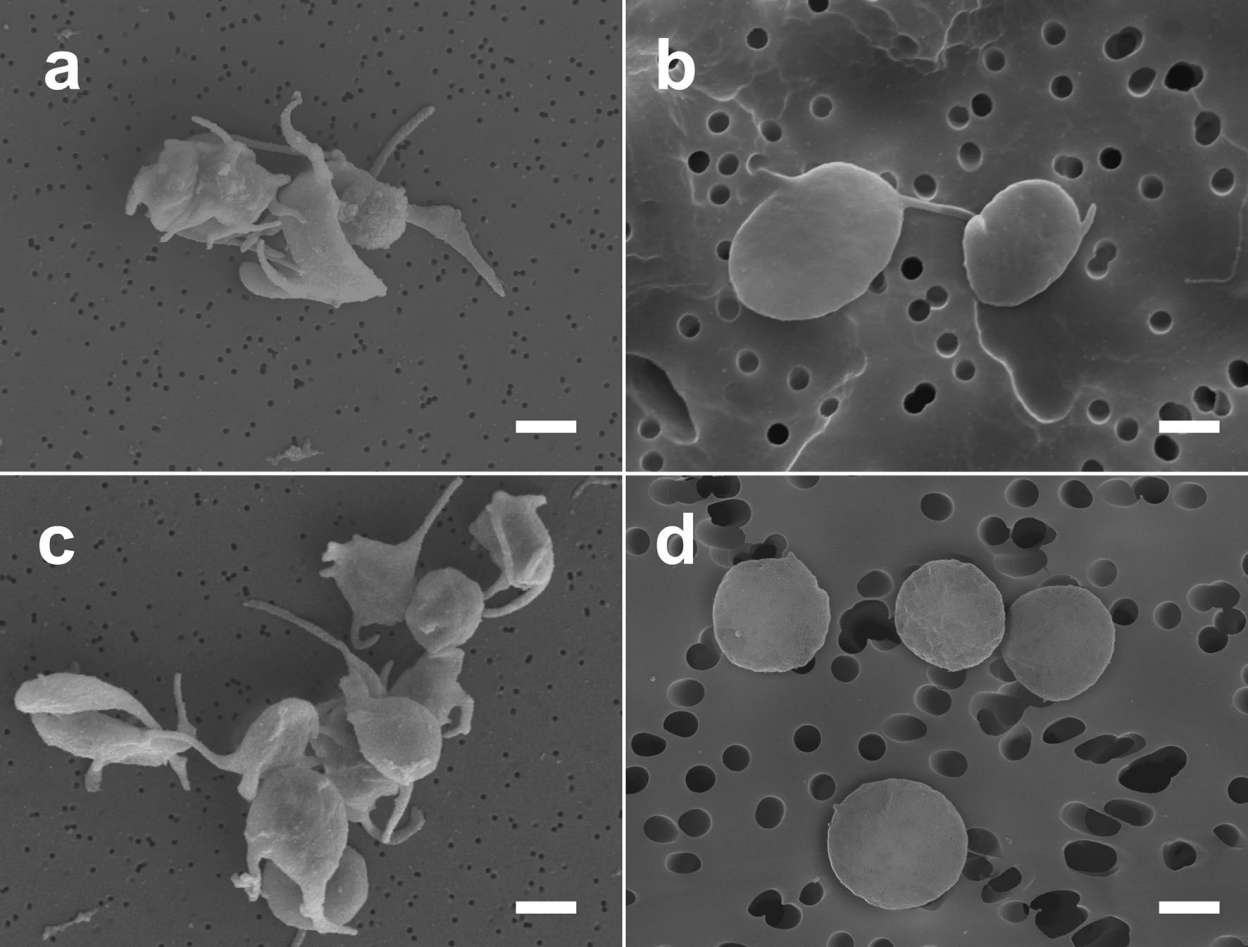
Representative scanning electron micrographs of activated (**a,c**) and resting platelets (**b,d**). Platelets isolated from the blood of COVID-19 patients (**a**–**c**) and a healthy donor (**d**). Magnification bars: 1 µm.

Scanning electron microscopy of platelets isolated from the blood of COVID-19 patients also showed some morphological signs of the background platelet activation in all the samples examined. Activated platelets lost their discoid form, shrank and formed multiple filipodia (Fig. 4a,c). The percentage of activated platelets in the quiescent COVID-19 blood samples, as assessed by the platelet morphology, varied but did not exceed 32% in any patient, while the vast majority of platelets analyzed (up to 92%) were resting or had only minimal signs of activation. They maintained a discoid shape with one or two short filopodia (Fig. 4b), while fully resting non-activated platelets had no filopodia (Fig. 4d).

The results obtained indicate that platelets are involved in the pathogenesis of COVID-19 and undergo definitive, yet moderate, alterations, both quantitative (mild thrombocytopenia) and qualitative (surface exposure of phosphatidylserine, expression and secretion of P-selectin, reduced expression of active integrin αIIbβ3 in response to stimulation, and morphological signs of cell activation). Phosphatidylserine exposure in resting platelets confirms that the background platelet activation is skewed towards procoagulant activity, leading to enhanced thrombin generation. Overall, the signs of the background platelet activation correlate directly with the laboratory markers of inflammation. Since flow cytometry and scanning electron microscopy of platelets were performed in relatively small subsets of patients, these data should be interpreted with caution.

### Association of thromboelastography and routine hemostatic tests with manifestations of COVID-19

According to thromboelastograms (TEGs), COVID-19 patients had longer reaction time (*R*) (Table 2, Fig. S3). This result corresponds to the prolonged lag time in the thrombodynamics assay (Table 1a), which, in turn, resembles the prothrombin time, since in both tests clotting is triggered with tissue factor. Notably, the clotting was slower in fatal cases (median 6.7 min, IQR 6; 7.2) compared to the patients who survived (5.7, IQR 4.8; 6.8; p < 0.01). Despite the delay in thrombin generation, patients’ samples had accelerated fibrin polymerization (*K*) and increased maximal amplitude (*MA*) (Table 2, Fig. S3). The maximal amplitude in TEG in the absence of platelets is a measure of greater clot stiffness that reflects the larger amount of fibrin and altered clot structure determined by hyperfibrinogenemia. These data show that in COVID-19, there is a link between hyperfibrinogenemia, increased clot stiffness, and impaired contraction revealed in this study.

In COVID-19 patients, hemostatic tests showed increased levels of D-dimer and prolongation of prothrombin time coupled with an increase of INR (Table 2). Patients with moderate severity had lower levels of D-dimer (median 671 ng/ml, IQR 414; 1250), compared with patients who had a severe form of the disease (1134 ng/ml, IQR 502; 2111; p < 0.05). In addition, the D-dimer levels had a weak, yet significant positive correlation with the leukocyte counts (r = 0.14; p < 0.05), including neutrophils (r = 0.22; p < 0.01), suggesting a connection between fibrin deposition and inflammation. Remarkably, the D-dimer levels had an inverse correlation with hemoglobin (r = − 0.23; p < 0.01), hematocrit (r = − 0.21; p < 0.01) and erythrocyte counts (r = − 0.25; p < 0.001), suggesting a direct relation between the pro-thrombotic state and anemia, both of which might be caused by inflammation. The ethanol gelation test, which detects soluble fibrin-monomer complexes, was positive in 20% of all the COVID-19 patients tested, with a ~ twofold prevalence in severe cases (33%), compared to the patients who had a moderately severe form of the disease (16%; p < 0.01).

## Discussion

We investigated hemostatic disorders in patients with acute COVID-19 in relation to the clinical manifestations of the disease and laboratory signs of inflammation. Importantly, the patients were examined while receiving LMWH and anti-inflammatory drugs that might interfere with the results of laboratory tests and blunt clinical symptoms. Despite this limitation, the results obtained confirm that in moderate-to-severe COVID-19, the disease is accompanied by a coagulopathy that correlates with the clinical course and outcomes, and was proportional to the systemic inflammation.

Hemostatic disorders were revealed using traditional blood testing along with two relatively new techniques, namely thrombodynamics and kinetics of blood clot contraction. The thrombodynamics assay quantifies the spatial growth of a plasma clot triggered by surface-immobilized tissue factor^24^. The blood clot contraction assay is based on the dynamic registration of reducing clot size under the contraction of activated platelets^18^. These two complementary assays have been useful to study pro-thrombotic conditions of various etiologies^25,26^.

Our results confirm that coagulopathies in COVID-19 develop primarily as hypercoagulability and a pre-thrombotic state. This conclusion is based on the high D-dimer levels; pronounced and persistent hyperfibrinogenemia; the presence of soluble fibrin-monomer complexes; formation of tissue factor-independent spontaneous plasma clots; accelerated polymerization and increased clot strength in TEG. These laboratory changes indicate hypercoagulability that develops even against a background of LMWH. Since D-dimer is a fibrinolytic cleavage product of cross-linked fibrin, a high level of D-dimer is a marker of ongoing intra- or extravascular fibrin deposition that can be local, regional or diffuse^27^. Fibrin formation is also supported by the positive ethanol gelation test, mostly in patients with severe COVID-19.

Paradoxically, in some COVID-19 patients, the signs of hypercoagulability go together with apparent hypocoagulability detected by a prolonged prothrombin time, reduced clot growth rate in the thrombodynamics assay, and prolonged clotting time in TEG. Evidently, all the parameters indicating hypocoagulability, unlike the laboratory signs of hypercoagulability, are chronometric, i.e., they represented the clotting time. We noticed that the slowed clotting was associated strongly with high blood levels of CRP (Table 1e), a marker of inflammation. CRP possesses direct anticoagulant activity via binding and blocking procoagulant phospholipids^14–16,28–30^; therefore, the observed delay in the in vitro plasma clotting could be due to hyper-production of CRP over 10 mg/l^15^. Antiphospholipid antibodies, often formed in COVID-19^31^, have similar properties and may also induce false hypocoagulability in vitro^32^, while in vivo the antiphospholipid antibodies promote thrombosis via immune activation of endothelial cells and monocytes^33^. This assumption has been confirmed by an inverse relation between the level of anti-cardiolipin antibodies and the initial clot growth rate (Fig. S2). Thus, it is possible that in severe inflammation, decelerated clotting in the chronometric tests reflects a high level of CRP^14–16,28–30^ and/or antiphospholipid antibodies^31^, which can mask real hypercoagulability. Practically speaking, in COVID-19 the blood clotting assays may have limitations, and the prolonged clotting time should be considered with caution before making clinical decisions, including prophylactic or therapeutic administration of anticoagulants. A similar paradox between a phospholipid-dependent prolongation of the clotting time and an increased risk of thrombosis has been described in the antiphospholipid syndrome and attributed to lupus anticoagulant^34^.

Our findings support the existence of a tight link between thrombophilia and inflammation in COVID-19. The laboratory indicators of hypercoagulability in COVID-19 correlate with the overproduction of the inflammatory markers (interleukin-6 and -8, fibrinogen, CRP) and cells (neutrophilia and monocytosis). This observation is in line with the concept that the inflammatory “cytokine storm” leads to immunothrombosis via multiple mechanisms^35–37^. For example, interleukin-6 induces expression of tissue factor on monocytes, macrophages, and endothelial cells, while TNF-α and interleukin-1 suppress the activity of physiological anticoagulants^38^. There are other potential mechanisms of positive feedback between inflammation and prothrombotic conditions^39^, including suppressed fibrinolysis as a result of binding of the SARS-CoV-2 virus to the ACE2 enzyme followed by dysfunction of the renin-angiotensin system^40^.

As a methodological advancement, the clot contraction assay was used for the integral evaluation of thrombotic risk in COVID-19. This assay was previously shown to be highly sensitive to ongoing and imminent thrombosis^19,20,25,26,41–43^ due to pathological alterations in platelet functionality and blood composition^18–20,25,26,42^. In patients with COVID-19, a pronounced impairment of clot contraction was found, which correlated directly with the disease severity and fatal outcomes. A decrease in the extent and rate of clot contraction worsened in the presence of comorbidities, including hematologic neoplasms, ischemic heart disease and acute ischemic stroke. In the male patients with COVID-19, the parameters of contraction were significantly worse compared with females, indicating a higher risk of thrombotic complications in men and reflecting the more severe course of the disease in men described earlier^44,45^. The extent of clot contraction directly correlated with the quantity of platelets in the blood, as well as with laboratory signs of anemia and coagulopathy, including high D-dimer, which confirmed the pathophysiological significance of contraction of blood clots and thrombi and the utility of the clot contraction kinetics assay in COVID-19.

The extent of contraction affects strongly the structure and cellular composition of blood clots. In the clots with abnormally weak contraction, which has been detected in more than a half of the patients examined, the clots are highly porous, have very few compacted and deformed polyhedral erythrocytes (polyhedrocytes) and uniform distribution of fibrin. In contrast, in clots that undergo strong contraction, clot porosity is low, tessellated arrays of polyhedrocytes prevail, and fibrin is accumulated at the clot periphery, as described previously in well-contracted blood clots and ex vivo thrombi^21,46,47^. These results provide a structural basis for the pathogenic relationship of the impaired blood clot contraction with a high risk of thrombotic complications in COVID-19. Firstly, compaction/shrinkage of a blood clot or thrombus during contraction reduces occlusion of the vessel and improves the local blood flow. In contrast, when contraction is impaired, the thrombotic obstruction of a vessel is more pronounced, which disturbs the blood flow and aggravates ischemia^20^. Secondly, contracted clots and thrombi with a high density of fibrin fibers and an increased local/intrinsic concentration of endogenous fibrinolytic enzymes are lysed faster, while poorly contracted clots with sparse fibrin fibers are less sensitive to internal fibrinolysis and are more durable^48^. Thirdly, the extent of contraction determines the mechanical stability of a thrombus, i.e., the predisposition to embolization, such that thrombi with a lower extent of contraction are weaker and potentially more embologenic^19^, which may explain the higher risk of pulmonary embolism and cardioembolic ischemic strokes in COVID-19^49–51^. On the other hand, uncompacted intravital clots with large intercellular pores are more permeable and could be prone to bleeding; therefore, intravascular formation of hemostatic blood clots with impaired ability to contract does not prevent hemorrhages developed in severe cases of COVID-19^52^.

In summary, our results support the notion that moderate and severe forms of COVID-19 are associated with either a pro-thrombotic state or ongoing microthrombosis that does not reach the scale of disseminated intravascular coagulation (DIC). Despite a high D-dimer level, which was associated with DIC in deceased patients with COVID-19^49^, the observed hyperfibrinogenemia in combination with moderate thrombocytopenia and insignificantly prolonged clotting time do not provide evidence for consumption coagulopathy and DIC. Obviously, in patients with moderate and severe, but not critical COVID-19, elevated D-dimer is a sign of local or regional (micro)thrombosis rather than DIC. If DIC develops in COVID-19, it occurs at the pre-terminal stage and underlies progressive multiple organ failure^53–55^.

Hence, COVID-19 should be considered as a (pre)thrombotic condition. The inflammation caused by SARS-CoV-2 is strongly associated with thrombophilia, so patients with COVID-19 should be considered at high risk of thrombosis. Unfortunately, despite the standard thromboprophylaxis with LMWH, laboratory signs of hypercoagulability may persist, and the incidence of thrombotic complications in COVID-19 remains high^7^. Therefore, monitoring the status of hemostasis is a source of important information necessary for the proper management of COVID-19.

## Conclusions

In acute COVID-19, hemostatic disorders that were revealed by extensive blood testing correlated with clinical manifestations of the disease, despite continuous LMWH and immunosuppressive therapy. About ¾ of the patients with COVID-19 had signs of hypercoagulability and pre-thrombosis that correlated directly with the production of systemic inflammation markers and the severity of the disease, including fatal outcomes. The most informative indicators of hypercoagulability were the levels of fibrinogen and D-dimer; the formation of tissue factor-independent spontaneous clots and high optical density of the clot in the thrombodynamics assay; the increased mechanical strength of the clot in TEG, and the impaired contraction kinetics of blood clots. The contraction of blood clots was most profoundly impaired in the severe cases and especially in those with fatal outcomes as well as in the presence of prothrombotic concomitant diseases, such as hematologic neoplasms, ischemic heart disease and acute cerebrovascular pathology. The suppressed blood clot contraction correlated directly with other signs of coagulopathy, including high D-dimer, which confirmed the pathophysiological significance of normal and impaired contraction of blood clots in COVID-19. A decrease in the extent and rate of clot contraction can be explained by moderate thrombocytopenia in combination with platelet dysfunction (reduced contractility) and hyperfibrinogenemia, which led to the formation of fibrin-rich strong clots that were resistant to compression. Meanwhile, the structure and cellular composition of blood clots and thrombi depended on the extent of contraction, such that poorly contracted clots were characterized by a high porosity, low content of compressed polyhedral erythrocytes (polyhedrocytes) and an even distribution of fibrin throughout the clot. Prolonged clotting tests combined with high levels of CRP and antiphospholipid antibodies, which are active in vitro anticoagulants, may mask the true hypercoagulability. In the absence of laboratory signs of consumption coagulopathy, the hemostatic disorders in COVID-19 are distinct from septic DIC and rather comprise potential or ongoing local or regional microthrombosis. The aggregate of data obtained confirms the important pathophysiologic role of (pro)thrombotic changes in COVID-19 and substantiates the importance of laboratory monitoring of hemostasis. Importantly, despite the use of LMWH, in most of the COVID-19 patients examined continuous activation of the hemostasis system persisted with a high risks of thrombotic complications that require personalized thromboprophylaxis.

## Methods

### Clinical material and ethics declaration

255 patients with acute COVID-19 were enrolled in the study, and were hospitalized in the City Hospital No. 16, Kazan, Russian Federation. The study was approved by the Ethics Committee of Kazan Federal University (reference #27 as of December 28, 2020) and written informed consent was obtained from the patients. All examinations were performed in accordance with the approved guidelines. The study was performed following the Declaration of Helsinki. All data from patients were treated anonymously. The investigation data were saved in an electronic database with restricted access.

The inclusion criteria were the following: age > 18 years; SARS-CoV-2 infection confirmed by PCR of the viral RNA; moderate or severe course of the disease; lung damage confirmed by a CT scan. The exclusion criteria were the age < 18 years and other concomitant infections (HIV, hepatitis B and C). All the patients received therapy that included immunosuppressive drugs and LMWH. The vast majority of patients (90%) received LMWH in a prophylactic or therapeutic dose except the patients with a tendency to bleed due to severe thrombocytopenia associated with neoplasms of hematopoietic or lymphoid tissues. The prophylactic dose of enoxaparin was 0.5 mg/kg once daily (standard) or twice daily (intermediate), and the therapeutic dose was 1.0 mg/kg twice daily^56^. Blood was drawn after overnight fasting and 12 h (if LMWH was administered twice daily) or 24 h (if LMWH was administered once daily) after the last subcutaneous injection of LMWH, i.e., with residual anticoagulant activity of heparin^57^. The criteria for administration of the standard, intermediate prophylactic or therapeutic doses of LMWH to COVID-19 patients were as follows: all hospitalized patients without severe thrombocytopenia were initially prescribed a standard prophylactic dose of LMWH corrected for a body weight. In case of progressive hypercoagulability assessed by growing levels of D-dimer and fibrinogen, the dosages of LMWH were increased up to the high prophylactic (if fibrinogen was ≤ 6.5 g/l and/or D-dimer ≤ 1500 ng/ml) or therapeutic (if fibrinogen reached > 6.5 g/l and/or D-dimer > 1500 ng/ml)^53^. None of the patients included in the study were on oral anticoagulants or antiplatelet drugs.

General clinical characteristics of the patients examined are summarized in Table S7. The control group included 60 healthy donors, 25 (42%) men and 35 (58%) women aged 24 to 87 years (average age 62 years). The main and control groups were matched by age and gender.

### Blood collection and processing

Blood was collected in vacutainers containing 3.2% trisodium citrate 9:1 by volume (S-Monovette, Sarstedt, Germany) and divided into 2 samples. The first sample of citrated blood was used for the clot contraction assay. The second blood sample was centrifuged at 200 g for 10 min at room temperature; platelet-rich plasma (PRP) was collected and used for flow cytometry and scanning electron microscopy of platelets. A portion of PRP was centrifuged at 2000×*g* for 10 min to obtain platelet-poor plasma (PPP) for clotting tests; a part of PPP was centrifuged at 10,000×*g* for 5 min to obtain platelet-free plasma (PFP) used for the thrombodynamics assay and thromboelastography. A sample of whole non-stabilized blood was mixed with a silicate activator to obtain serum for biochemical tests. Another blood sample was stabilized with K_3_-EDTA (1.6 mg/ml final concentration) and used for hematological tests. The blood was processed at room temperature and used within 4 h after collection.

### Thrombodynamics assay (fibrin clot propagation in space and time)^58^

**Figure 5:**
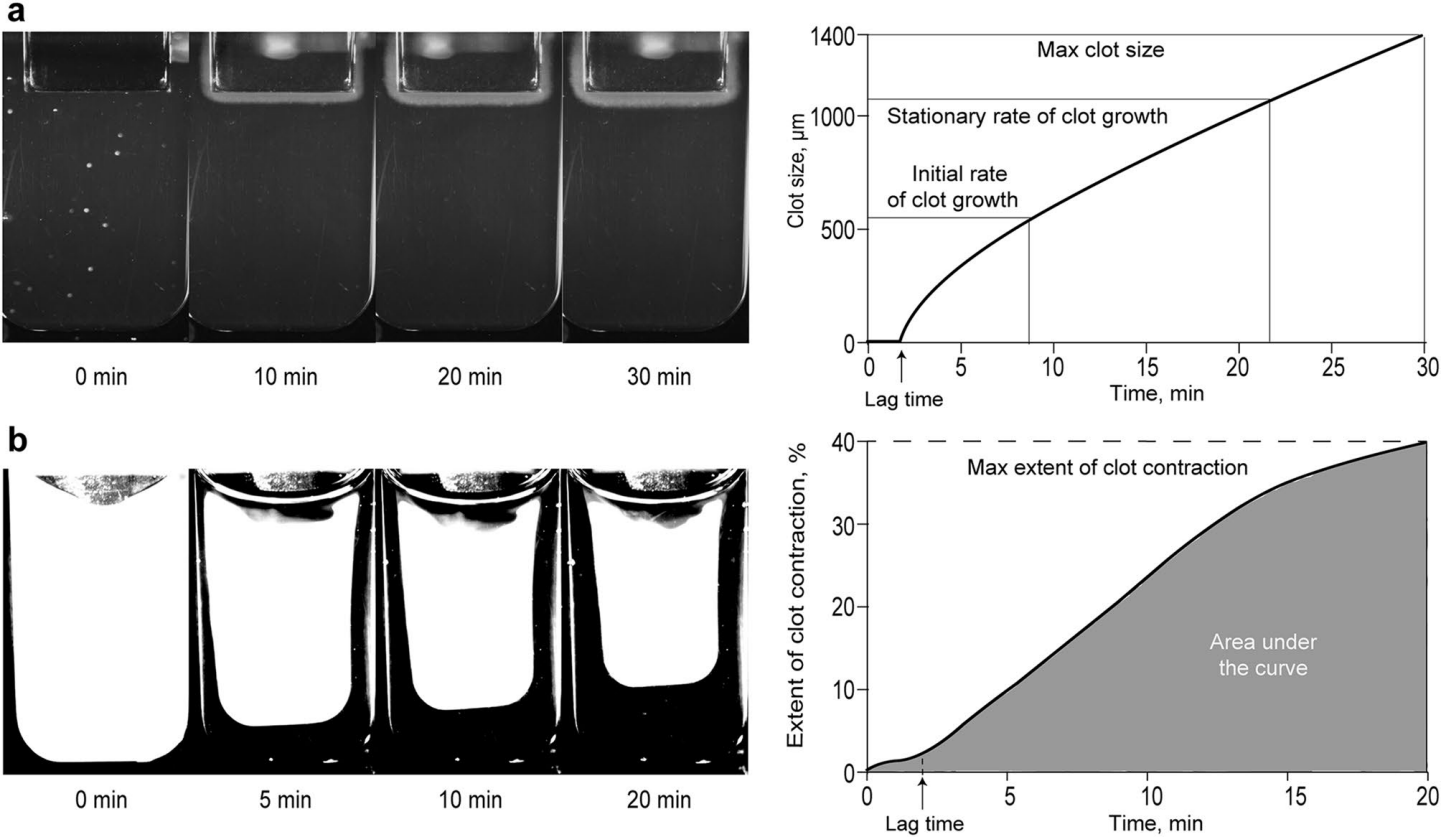
The optical system used to quantify the spatial growth of plasma clots on a tissue-factor-coated insert (thrombodynamics) (**a**) and the kinetics of contraction (retraction) of blood clots (**b**). On the left are images of cuvettes containing a growing (**a**) or contracting (**b**) clot and on the right are the output kinetics curves with the corresponding parameters.

The thrombodynamics assay was performed in 200 patients and 20 healthy donors. PFP was mixed with contact phase inhibitors and calcium acetate. An activating insert coated with tissue factor was immersed into the plasma to mimic the damaged surface. The fibrin clot started growing from the insert into the bulk of the plasma sample and fibrin formation was recorded by the Thrombodynamics Analyzer (HemaCore, Russia) (Fig. 5a). The following parameters were calculated from serial images of the growing clot: (1) lag time—the time required to start the formation of fibrin from the moment of contact of the plasma with the activating surface; (2) the initial growth rate of the clot—the median growth rate of the clot calculated between 2 and 6 min after the onset of clot growth; (3) the stationary growth rate of the clot—the median growth rate of the clot calculated between 15 and 25 min after the onset of clot growth; (4) the size of the fibrin clot after 30 min following the contact of plasma with the activator insert; (5) clot density—an optical parameter equal to the intensity of light scattering by the fibrin clot, proportional to the density of the fibrin network.

### Kinetics of blood clot contraction^18^

The blood clot contraction assay was performed in 215 patients and 40 healthy donors using the same registration device, the Thrombodynamics Analyzer. A plastic cuvette 12 × 7 × 1 mm was pre-rinsed with 4% Triton X-100 in 150 mM NaCl to prevent adhesion of fibrin to the walls. In a separate plastic test tube, CaCl_2_ (2 mM final) and human thrombin (1 U/ml final) were added to initiate blood coagulation and activate platelets. The activated blood (80 μl) was transferred into the cuvette preheated to 37 °C. Registration of the clot size was performed every 15 s for 20 min after the addition of thrombin (Fig. 5b). The serial images of the clot were converted into a kinetic curve, from which the following parameters were calculated: (1) the extent of contraction, reflecting the extent of clot compression (in percent) relative to its initial size after 20 min of registration; (2) lag time—the time during which the clot reaches 95% of its initial size; (3) the area under the kinetic curve, which reflects the amount of mechanical work on clot compression performed by the platelets. (4) The average contraction velocity—the compression ratio of clot (in percent) per unit of time. The border of normal and reduced extent of clot contraction determined based on the specificity and sensitivity of this assay was at the level of 41%^41^. To exclude potential effects of residual LMWH activity, blood samples with reduced extent of contraction were re-tested after pretreatment with protamine sulfate (15 µg/ml final, 5 min, 37 °C) to neutralize heparin.

### Flow cytometry of platelets

Flow cytometry was performed in 40 patients and 20 healthy donors to assess platelet functionality by the expression of P-selectin (CD62P) and active integrin αIIbβ3 before and after stimulation. A PRP sample diluted with Tyrode’s buffer (50 µl containing 400,000 cells) was incubated for 10 min with antibodies against CD62P labeled with phycoerythrin (0.045 μg/ml)(BD Biosciences, USA) and with PAC-1 antibodies against the active form of the integrin αIIbβ3 labeled with fluorescein 5-isothiocyanate (1 μg/ml) (BD Biosciences, USA). Platelet activator TRAP-6 (50 μM) was added to PRP, incubated for 3 and 10 min at room temperature, after which platelets were analyzed using a CytoFLEX flow cytometer (Beckman Coulter, USA). Platelets were gated based on their size (forward scatter, FSC) and granularity (side scatter, SSC); 5000 platelets were counted in each sample.

### Scanning electron microscopy of platelets

PRP samples from the blood of 25 patients were diluted tenfold with Tyrode's buffer (4 mM HEPES; 135 mM NaCl; 2.7 mM KCl; 2.4 mM MgCl_2_; 3.3 mM NaH_2_PO_4_; 5.6 mM d-glucose; 0.3% bovine serum albumin, pH 7.4), after which platelets were fixed with glutaraldehyde (2%) in saline for 90 min at room temperature. The cells were deposited by centrifugation (500×*g*, 3 min) on a polycarbonate filter with a pore size of 0.1 or 0.4 μm, washed with phosphate-buffered saline, then dehydrated with 30–100% ethanol. The samples were treated with hexamethyldisilazane (HMDS), then air-dried. Dry samples were coated with gold and palladium on Polaron e5100 or Quorum Q150T ES sputter coaters, followed by imaging with a scanning electron microscope (Quanta 250FEG, FEI, USA).

### Scanning electron microscopy of blood clots

Clots from whole citrated blood of 24 patients formed as a result of thrombin-induced blood clotting and contraction were rinsed with saline and fixed in 2% glutaraldehyde in saline for 90 min at room temperature. The clots were cut-open and dehydrated by increasing concentrations of ethanol from 30 to 100%, after which alcohol was replaced with HMDS and the samples were air-dried (see the previous paragraph). The samples were oriented with the sliced surface up to visualize the clot interior, sputter-coated with a mixture of gold and palladium (Polaron e5100) and imaged using a Quanta 250FEG scanning electron microscope (FEI, USA).

### Thromboelastography

Thromboelastography was performed using the TEG 5000 (Haemoscope, USA) instrument in 215 patients and 40 healthy donors. PFP was pre-incubated with kaolin (0.01 ng/ml final) at room temperature for 5 min. Kaolin-activated PFP (340 μl) was transferred into a TEG cuvette pre-warmed to 37 °C with CaCl_2_ (20 μl) to make 20 mM final concentration. The following parameters were determined by TEG: *R*—reaction time, i.e., time from the initiation of clotting to the onset of fibrin formation; *K*—the fast phase of fibrin polymerization, i.e., time to increase the TEG amplitude from 2 to 20 mm; *α*—the slope of a TEG curve, characterizing the rate of clot formation; *MA* is the maximum TEG amplitude, which reflects the mechanical strength of the clot formed.

### Coagulation, hematological, and biochemical tests

In all 235 patients and 50 healthy donors, the following coagulation tests were performed: prothrombin time and international normalized ratio (INR), the fibrinogen Clauss assay, D-dimer test using an ELISA kit (Siemens, USA) and Immulite 2000XPi analyzer (Siemens, USA). The ethanol gelation test comprised addition of 0.15 ml 50% ethanol to 0.4 ml of platelet-free citrate plasma at room temperature; in 10 min the appearance of turbidity or sediment was a sign of soluble fibrin-monomer complexes and thrombinemia^59^. Hematological analysis was performed on a BC-3600 analyzer (Mindray, China). The levels of interleukins-6 and -8 in the blood serum of 78 patients were determined using ELISA kits from Vector-Best (Russia) and a StatFax 2100 reader. Soluble sP-selectin in the serum of 78 patients was determined with an ELISA kit (Invitrogen, USA) and Sunrise microplate reader (Tecan, Switzerland). Total anti-cardiolipin antibodies (IgG, IgM and IgA) were determined in the blood serum of 84 patients using an ELISA kit (OriGene, USA) and a StatFax 2100 reader.

### Statistical analysis

Statistical analysis was performed using the GraphPad Prism 8 and RStudio software packages. The D'Agostino-Pearson, Kolmogorov–Smirnov and Shapiro–Wilk tests were used to assess the normality of the sample distribution. Statistical differences for paired comparisons were assessed using the Mann–Whitney nonparametric U-test. For multivariate analysis, the Kruskal–Wallis test was used followed by pairwise comparisons with the Dunn test. Correlation analysis was performed using the Spearman's rank correlation coefficient. The chi-square test was used to compare categorical variables. The level of statistical significance was 95% (p < 0.05).

## Supplementary Information

Supplementary Information.

## Data Availability

All data generated or analyzed during this study are included in this published article (and its Supplementary Information files).

## References

[1] Tal S, Spectre G, Kornowski R, Perl L (2020). Venous thromboembolism complicated with COVID-19: What do we know so far?. Acta Haematol..

[2] Cui S, Chen S, Li X, Liu S, Wang F (2020). Prevalence of venous thromboembolism in patients with severe novel coronavirus pneumonia. J. Thromb. Haemost..

[3] Helms J (2020). High risk of thrombosis in patients with severe SARS-CoV-2 infection: A multicenter prospective cohort study. Intensive Care Med..

[4] Middeldorp S (2020). Incidence of venous thromboembolism in hospitalized patients with COVID-19. J. Thromb. Haemost..

[5] Paranjpe I (2020). Association of treatment dose anticoagulation with in-hospital survival among hospitalized patients with COVID-19. J. Am. Coll. Cardiol..

[6] Thachil J (2020). ISTH interim guidance on recognition and management of coagulopathy in COVID-19. J. Thromb. Haemost..

[7] Hadid T, Kafri Z, Al-Katib A (2020). Coagulation and anticoagulation in COVID-19. Blood Rev..

[8] Leentjens J, van Haaps TF, Wessels PF, Schutgens REG, Middeldorp S (2021). COVID-19-associated coagulopathy and antithrombotic agents: Lessons after 1 year. Lancet Haematol..

[9] Wang J (2020). Dysfunctional coagulation in COVID-19: From cell to bedside. Adv. Ther..

[10] Nicolai L (2020). Immunothrombotic dysregulation in COVID-19 pneumonia is associated with respiratory failure and coagulopathy. Circulation.

[11] Iba T, Levy JH, Levi M, Thachil J (2020). Coagulopathy in COVID-19. J. Thromb. Haemost..

[12] Wool GD, Miller JL (2021). The impact of COVID-19 disease on platelets and coagulation. Pathobiology.

[13] Bowles L (2020). Lupus anticoagulant and abnormal coagulation tests in patients with Covid-19. N. Engl. J. Med..

[14] van Rossum AP, Vlasveld LT, van den Hoven LJM, de Wit CWM, Castel A (2012). False prolongation of the activated partial thromboplastin time (aPTT) in inflammatory patients: Interference of C-reactive protein. Br. J. Haematol..

[15] Devreese KMJ, Verfaillie CJ, De Bisschop F, Delanghe JR (2015). Interference of C-reactive protein with clotting times. Clin. Chem. Lab. Med..

[16] Thaker A, Chandler W (2017). Prolongation of PTT by CRP is magnified in the setting of heparin and warfarin therapy. Am. J. Clin. Pathol..

[17] Zlatko D (2015). The Cytokines of the Immune System: The Role of Cytokines in Disease Related to Immune Response.

[18] Tutwiler V (2016). Kinetics and mechanics of clot contraction are governed by molecular and cellular blood composition. Blood.

[19] Peshkova AD (2018). Reduced contraction of blood clots in patients with venous thromboembolism is a possible thrombogenic and embologenic mechanism. TH Open..

[20] Tutwiler V (2017). Blood clot contraction is impaired in acute ischemic stroke. Arterioscler. Thromb. Vasc. Biol..

[21] Cines DB (2014). Clot contraction: Compression of erythrocytes into tightly packed polyhedra and redistribution of platelets and fibrin. Blood.

[22] Tutwiler V (2018). Shape changes of erythrocytes during blood clot contraction and the structure of polyhedrocytes. Sci. Rep..

[23] Caine GJ, Blann AD (2003). Soluble P-selectin should be measured in citrated plasma, not in serum. Br. J. Haematol..

[24] Tuktamyshov R, Zhdanov R (2015). The method of in vivo evaluation of hemostasis: Spatial thrombodynamics. Hematology.

[25] Peshkova AD (2019). Premorbid hemostasis in women with a history of pregnancy loss. Thromb. Haemost..

[26] Peshkova AD (2020). Accelerated spatial fibrin growth and impaired contraction of blood clots in patients with rheumatoid arthritis. Int. J. Mol. Sci..

[27] Matsuo T, Kobayashi H, Kario K, Suzuki S (2000). Fibrin D-dimer in thrombogenic disorders. Semin. Thromb. Hemost..

[28] Liu J (2018). The analysis of false prolongation of the activated partial thromboplastin time (activator: silica): Interference of C-reactive protein. J. Clin. Lab. Anal..

[29] Schouwers SME, Delanghe JR, Devreese KMJ (2010). Lupus anticoagulant (LAC) testing in patients with inflammatory status: Does C-reactive protein interfere with LAC test results?. Thromb. Res..

[30] Ruinemans-Koerts J, Ahmed-Ousenkova YM, Kaasjager HAH, Hendriks-van Wijhe C, Hovens MMC (2015). When to screen for lupus anticoagulant? Influence of testing during acute phase and consequences for clinical practise. Lupus.

[31] Zhang Y (2020). Coagulopathy and antiphospholipid antibodies in patients with Covid-19. N. Engl. J. Med..

[32] Dalal KS, Bridgeman MB (2017). Cardiovascular drugs. Nursing (Lond).

[33] Velásquez M, Rojas M, Abrahams VM, Escudero C, Cadavid ÁP (2018). Mechanisms of endothelial dysfunction in antiphospholipid syndrome: Association with clinical manifestations. Front. Physiol..

[34] Molhoek JE, de Groot PG, Urbanus RT (2018). The lupus anticoagulant paradox. Semin. Thromb. Hemost..

[35] Wu W (2020). Clinical features of patients infected with 2019 novel coronavirus in Wuhan, China. Lancet.

[36] Ranucci M (2020). The procoagulant pattern of patients with COVID-19 acute respiratory distress syndrome. J. Thromb. Haemost..

[37] Mangalmurti N, Hunter CA (2020). Cytokine storms: Understanding COVID-19. Immunity.

[38] Mehta P (2020). COVID-19: Consider cytokine storm syndromes and immunosuppression. Lancet.

[39] Gu SX (2021). Thrombocytopathy and endotheliopathy: Crucial contributors to COVID-19 thromboinflammation. Nat. Rev. Cardiol..

[40] Sriram K, Insel PA (2021). Inflammation and thrombosis in COVID-19 pathophysiology: Proteinase-activated and purinergic receptors as drivers and candidate therapeutic targets. Physiol. Rev..

[41] Evtugina NG, Peshkova AD, Pichugin AA, Weisel JW, Litvinov RI (2020). Impaired contraction of blood clots precedes and predicts postoperative venous thromboembolism. Sci. Rep..

[42] Le Minh G (2018). Impaired contraction of blood clots is a novel prothrombotic mechanism in systemic lupus erythematosus. Clin. Sci. (Lond.).

[43] Peshkova AD, Malyasev DV, Bredikhin RA, Le Minh G, Litvinov RI (2016). Contraction of blood clots is impaired in deep vein thrombosis. BioNanoScience.

[44] Takahashi T (2020). Sex differences in immune responses that underlie COVID-19 disease outcomes. Nature.

[45] Gebhard C (2020). Impact of sex and gender on COVID-19 outcomes in Europe. Biol. Sex Differ..

[46] Chernysh IN (2020). The distinctive structure and composition of arterial and venous thrombi and pulmonary emboli. Sci. Rep..

[47] Khismatullin RR (2020). Quantitative morphology of cerebral thrombi related to intravital contraction and clinical features of ischemic stroke. Stroke.

[48] Tutwiler V (2019). Blood clot contraction differentially modulates internal and external fibrinolysis. J. Thromb. Haemost..

[49] Tang N, Li D, Wang X, Sun Z (2020). Abnormal coagulation parameters are associated with poor prognosis in patients with novel coronavirus pneumonia. J. Thromb. Haemost..

[50] Danzi GB, Loffi M, Galeazzi G, Gherbesi E (2020). Acute pulmonary embolism and COVID-19 pneumonia: A random association?. Eur. Heart J..

[51] Bonow RO, Fonarow GC, O’Gara PT, Yancy CW (2020). Association of coronavirus disease 2019 (COVID-19) with myocardial injury and mortality. JAMA Cardiol..

[52] Li JY (2021). Thrombo-COVID-19 Collaborative. Clinical characteristics and risk factors for symptomatic venous thromboembolism in hospitalized COVID-19 patients: A multicenter retrospective study. J. Thromb. Haemost..

[53] Ministry of Health of the Russian Federation. *Temporary Guidelines “Prevention, Diagnosis and Treatment of the New Coronavirus Infection (COVID-19)”, Version 10.* 261. (Ministry of Health of the Russian Federation, 2021) (**In Russ.**).

[54] Terpos E (2020). Hematological findings and complications of COVID-19. Am. J. Hematol..

[55] Al-Samkari H (2020). COVID-19 and coagulation: Bleeding and thrombotic manifestations of SARS-CoV-2 infection. Blood.

[56] Royal College of Obstetricians and Gynaecologists (RCOG) (2015). Thrombosis and Embolism During Pregnancy and the Puerperium: Acute Management. Green-Top Guideline No. 37b.

[57] Alban S, Welzel D, Hemker HC (2002). Pharmacokinetic and pharmacodynamic characterization of a medium-molecular-weight heparin in comparison with UFH and LMWH. Semin. Thromb. Hemos..

[58] Sinauridze EI (2018). Thrombodynamics, a new global coagulation test: Measurement of heparin efficiency. Talanta.

[59] Breen FA, Tullis JL (1969). Ethanol gelation test improved. Ann. Intern. Med..

