# Supplementary material for: Altered platelet and coagulation function in moderate-to-severe COVID-19
Source: Sci Rep. 2021 Aug 11;11:16290. doi: 10.1038/s41598-021-95397-6 (PMC8357814; doi:10.1038/s41598-021-95397-6)
Supplement: Supplementary file 1 — Supplementary Information. [file 41598_2021_95397_MOESM1_ESM.pdf]

Supplementary Information

Altered platelet and coagulation function in moderate-to-severe COVID-19

Rustem I. Litvinov[1,2], Natalia G. Evtugina[2], Alina D. Peshkova[2], Svetlana I. Safiullina[2,3], Izabella A. Andrianova[2], Alina I. Khabirova[2], Chandrasekaran Nagaswami[1], Rafael R. Khismatullin[2], Svetlana S. Sannikova[4], John W. Weisel[1,*]

[1]Department of Cell and Developmental Biology, University of Pennsylvania School of Medicine, Philadelphia, Pennsylvania, USA
[2]Institute of Fundamental Medicine and Biology, Kazan (Volga region) Federal University, Kazan, Russian Federation
[3]Medical Center "Aibolit", Kazan, Russian Federation
[4]City Hospital No.16, Kazan, Russian Federation

*Correspondence:
Professor John W. Weisel
Department of Cell and Developmental Biology
University of Pennsylvania Perelman School of Medicine
421 Curie Blvd., BRB II/III, Room 1153
Philadelphia, PA 19104 USA
Tel.: +1-215-898-3573
E-mail: weisel@pennmedicine.upenn.edu
ORCiD: https://orcid.org/0000-0002-9628-257X

Supplementary Tables

Table S1. Correlation analysis of the parameters of thrombodynamics and other laboratory parameters (significant Spearman's coefficients)

Correlated parameters	*Parameters of thrombodynamics*				
	Lag time	*Initial rate of clot growth*	*Stationary rate of clot growth*	*Clot size*	*Clot density*
Prothrombin time	-0.41**	-	-0.33*	-0.38**	-
ESR	-	-	-	-	0.48***
Monocyte count	-	-	-	0.21**	-
AST	-	-	-	-	0.31***
Total protein	0.26**	-	-	-	-
CRP	0.24**	-	-	-	0.26**
Interleukin-8	-	0.31**	-	0.25*	-

*p<0.05; **p<0.01; ***p<0.001

Table S2. Hemostatic parameters in COVID-19 patients receiving different LMWH doses

Parameters (in parenthesis – reference values)	*Standard prophylactic dose of LMWH (n=111)*	*High prophylactic dose of LMWH (n=96)*	*Therapeutic dose of LMWH (n=8)*	*p values*
Stationary rate of clot growth (20-29), μm/min	25.7 (19.7; 32.1)	23.2 (17.3; 31.4)	20.4 (9.2; 28.0)	*p*=0.29
Lag time (0.6-1.5), min	1.3 (1.1; 1.5)	1.3 (1.2; 1.6)	1.6 (1.3; 1.7)	*p*=0.03
Initial rate of clot growth (38-56), μm/min	55.3 (49.8; 59.7)	53.2 (49.7; 59.8)	45.5 (36.2; 55.1)	*p*=0.09
Clot size (800-1200), μm	1130 (969; 1299)	1023 (894; 1217)	922 (596; 1161)	*p*=0.03
Clot density (15000-32000), a.u.	28740 (24911; 31341)	29944 (24836; 32573)	28330 (25398; 33587)	*p*=0.35
TEG:R, min	5.8 (4.7; 6.8)	5.8 (5.0; 6.9)	5.4 (4.9; 7.1)	*p*=0.80
TEG: MA, mm	37.9 (33.3; 43.4)	40.5 (34.6; 44.9)	38.4 (28.5; 48.9)	*p*=0.22
Extent of clot contraction, %	41 (37; 43)	41 (36; 43)	41 (35; 45)	*p*=0.95
D-dimer, ng/ml	699 (435; 1587)	694 (396; 1295)	2060 (909; 3576)	*p*=0.08
INR (0-1.3)	1.0 (0.9; 1.1)	1.0 (1.0; 1.1)	1.1 (1.0; 1.1)	*p*=0.29
Prothrombin ratio (80-105), %	98 (93; 103)	98 (92; 100)	94 (90; 100)	*p*=0.40
Fibrinogen (2.0-4.0), g/l	4.6 (3.7; 6.0)	5.5 (3.9; 5.9)	5.5 (3.9; 6.6)	*p*=0.15
Platelet count (180-360), $\times 10^9$/l	222 (159; 286)	242 (167; 308)	215 (153; 266)	*p*=0.30

The results are presented as median and interquartile range (25th and 75th percentiles).
The Kruskal-Wallis test.

Table S3. Hematologic parameters in patients with COVID-19 and healthy donors

Parameters (in parentheses - reference values)	*Patients with COVID-19 (n=235)*	*Healthy subjects (n=50)*
Hemoglobin (110-160), g/l	126 (110; 137)**	146 (135; 153)
Hematocrit (37-54), %	39 (34; 43)	41 (36; 43)
ESR (2-5), mm/h	32 (18; 45)**	7 (5; 12)
Red blood cells (3.9-4.7), $\times 10^{12}$/l	4.3 (3.8; 4.7)*	4.6 (4.3; 4.9)
Mean corpuscular volume, MCV (80-100), fl	89 (86; 93)**	86 (84; 88)
Mean corpuscular hemoglobin, MCH (27-34), pg	28.9 (27.6; 29.8)**	31.7 (29.8; 33.7)
Red blood cell distribution width, RDW (11-16), %	13.9 (13.4; 15.2)	13.9 (13.4; 14.6)
Mean corpuscular hemoglobin concentration, MCHC (320-360), g/l	321 (315; 326)**	366 (352; 386)
Platelet count (180-360), $\times 10^9$/l	225 (158; 292)*	247 (220; 300)
Mean platelet volume, MPV (6.5-12.0), fl	9.7 (9.0; 10.8)**	8.1 (7.5; 8.8)
Thrombocrit (0.108-0.282), %	0.228 (0.179; 0.287)	0.260 (0.200; 0.305)
Leukocyte count (4-10), $\times 10^9$/l	7.5 (5.2; 11.1)**	5.1 (4.2; 6.2)
Neutrophils (46-72), %	74.0 (64.7; 83.0)**	58.3 (54.6; 64.5)
Neutrophil count (2-7), $\times 10^9$/l	5.2 (3.4; 8.3)**	2.9 (2.2; 3.6)
Monocytes (3-11), %	5.2 (4.0; 8.0)	6.0 (5.1; 6.9)
Monocyte count (0.1-1.5), $\times 10^9$/l	0.4 (0.2; 0.7)*	0.3 (0.2; 0.4)
Lymphocytes (18-38), %	17 (10; 24)**	35 (29; 39)
Lymphocyte count (0.8-4.0), $\times 10^9$/l	1.1 (0.7; 1.7)**	1.7 (1.4; 2.3)
Eosinophils (0.5-5), %	1.0 (0; 3.0)	1.0 (0.8; 1.2)
Eosinophil count (0.02-0.30), $\times 10^9$/l	0.10 (0; 0.19)	0.10 (0.07; 0.17)
Basophils (0-1), %	0	0.6 (0.3; 0.7)
Basophil count (0-0.07), $\times 10^9$/l	0	0.04 (0.03; 0.05)

Results are presented as median and interquartile range (25th and 75th percentiles).
*p<0.01, **p<0.0001, Mann-Whitney U-test

Table S4. Correlation analysis of the kinetic parameters of clot contraction and other laboratory parameters (significant Spearman's coefficients)

Correlated parameters	*Parameters of blood clot contraction*			
	Extent of clot contraction	*Lag time*	*Area under the curve*	*Average velocity*
Platelet count	0.25***	-0.26***	0.31***	0.25***
Prothrombin time	-	0.35**	-0.33**	-
Thrombocrit	-	-	0.22*	0.24*
Mean corpuscular volume (MCV)	-0.24*	-	-	-0.22*
Mean corpuscular hemoglobin (MCH)	-0.29**	-	-	-0.25**

*p<0.05; **p<0.01; ***p<0.01

Table S5. Comparative characteristics of the structure and composition of clots from the blood of patients with COVID-19 with reduced (<41%) and normal (≥41%) extent of contraction

Structural elements	*Contraction <41% (n=17)*	*Contraction ≥41% (n=7)*
The relative volume occupied by each structural element		
RBCs	70% (60; 80)**	90% (85; 94)
Fibrin	15% (10; 20)**	5% (5;10)
Empty space	15% (10; 20)**	5% (3; 10)
The relative number (fraction) of RBCs with ascending degree of compression		
Biconcave RBCs	10% (10; 25)***	0
Mainly biconcave intermediate-shaped RBCs	35% (30; 45)**	10% (3; 20)
Mainly polyhedral intermediate-shaped RBCs	30% (20; 40)	20% (10; 30)
Polyhedral RBCs	15% (10; 20)***	70% (40; 80)

Results are presented as median and interquartile range (25th and 75th percentiles).
*p<0.05; **p<0.01; ***p<0.001; Mann-Whitney U-test

Table S6. Functional characterization of platelets in the blood of patients with COVID-19 compared to healthy subjects

	P-selectin			*Active integrin αIIbβ3*			*PS*
	Quiescent platelets	TRAP-activated platelets (3 min)	TRAP-activated platelets (10 min)	Quiescent platelets	TRAP-activated platelets (3 min)	TRAP-activated platelets (10 min)	Quiescent platelets
Patients with COVID-19 (n=20)	2.3 (1.2; 3.0)*	85 (77; 87)*	90 (88; 92)	3.3 (1.2; 4.5)	89 (85; 93)	91 (89; 94)**	6.3 (3.9; 11.2)**
Healthy subjects (n=10)	1.1 (0.6; 1.8)	73 (65; 81)	91 (88; 93)	1.8 (1.2; 2.0)	92 (88; 92)	95 (93; 96)	1.2 (0.7; 2.0)

The numbers represent the proportion (%) of platelets, expressing P-selectin, active integrin αIIbβ3 or phosphatidylserine (PS) before and after activation with TRAP; the results are presented as median and interquartile range (25th and 75th percentiles).
*$p<0.05$; **$p<0.01$; Mann-Whitney U-test

Table S7. Demographic and clinical characteristics of the COVID-19 patients enrolled in this study

Characteristics		*Number of patients (n=255)*
Gender	Men	115 (45%)
	Women	140 (55%)
Age, years	<65	97 (38%)
	≥65	158 (62%)
The severity of the disease	Moderate*	184 (72%)
	Severe*	71 (28%)
The extent of lung damage (CT scan)	Mild*	55 (22%)
	Moderate*	118 (46%)
	Severe*	82 (32%)
Duration of the disease at the time of examination	<7 days from onset of the disease	18 (7%)
	7-14 days from the onset of the disease	111 (44%)
	>14 days from onset of the disease	126 (49%)
Outcome of the disease	Favorable	237 (93%)
	Lethal	18 (7%)
Comorbidities and risk factors		
Neoplasms of hematopoietic or lymphoid tissues		7 (3%)
Hypertension		142 (56%)
Diabetes		62 (24%)
Coronary heart disease		47 (18%)
Acute myocardial infarction		19 (7%)
Cerebral ischemic stroke or transient cerebral ischemic attacks		26 (10%)
Phlebitis and thrombophlebitis of superficial vessels of lower extremities		10 (4%)
Smoking		20 (8%)
Obesity (BMI > 30 kg/m^2)		84 (33%)
Therapy		
Low molecular weight heparin	Standard prophylactic dose (enoxaparin 0.5 mg/kg once daily)	104 (41%)
	High prophylactic and therapeutic doses (enoxaparin 0.5 or 1.0 mg/kg twice daily)	151 (59%)
Glucocorticosteroids (dexamethasone, methylprednisolone, prednisolone)		241 (95%)
Interleukin-6 inhibitors (tocilizumab)		7 (3%)
Antiviral drugs (ingavirin, arbidol)		68 (27%)
Mucolytics (ACC, ambroxol)		124 (49%)
Hydroxychloroquine		84 (33%)
Mechanical ventilation		16 (6%)

*Defined by the Temporary guidelines “Prevention, diagnosis and treatment of the new coronavirus infection (COVID-19)” issued on February 9, 2021, by the Ministry of Health of the Russian Federation [ref. 53 in the main text].

Supplementary Figures

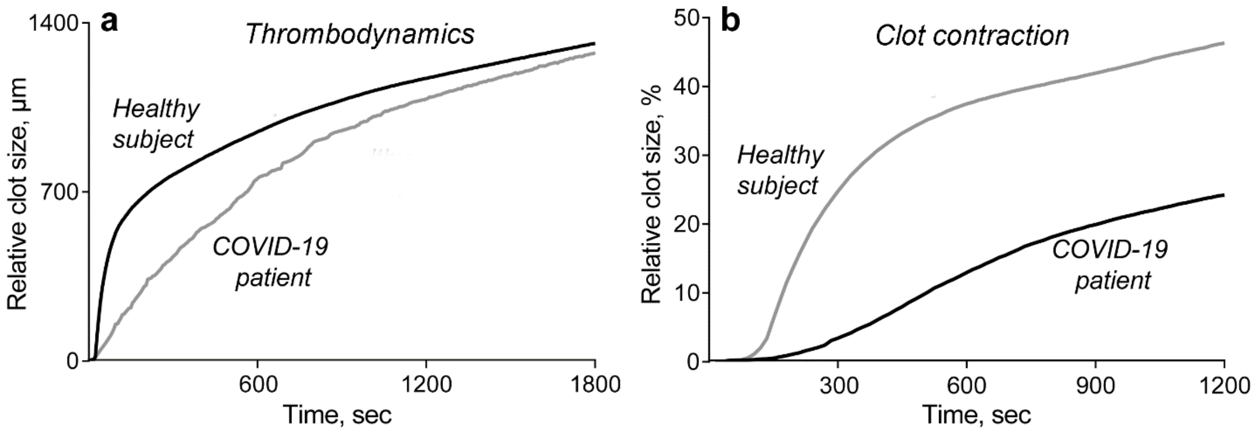

Figure S1. Characteristic examples of individual kinetic curves showing differential spatial clot growth in the thrombodynamics assay (**a**) and clot shrinkage in the clot contraction assay (**b**) in blood samples of a COVID-19 patient and a healthy subject.

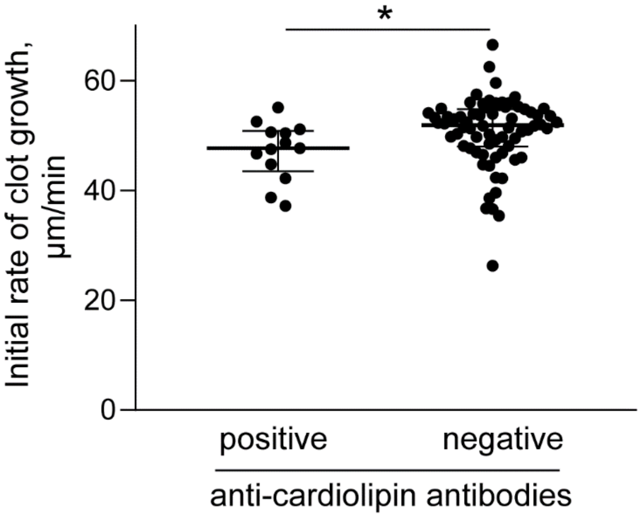

Figure S2. The initial rate of clot growth (thrombodynamics assay) in the subgroups of COVID-19 patients that had elevated (positive) and normal (negative) levels of the total anti-cardiolipin antibodies (IgG, IgM, IgA) in blood serum. According to the manufacturer (OriGene, USA), the positive test corresponds to >15 IPL-U/ml for IgG, >20 MPL-U/ml for IgM, and >20 APL-U/ml for IgA. Results are presented as median and interquartile range (25th and 75th percentiles); *$p<0.05$; Mann-Whitney U-test

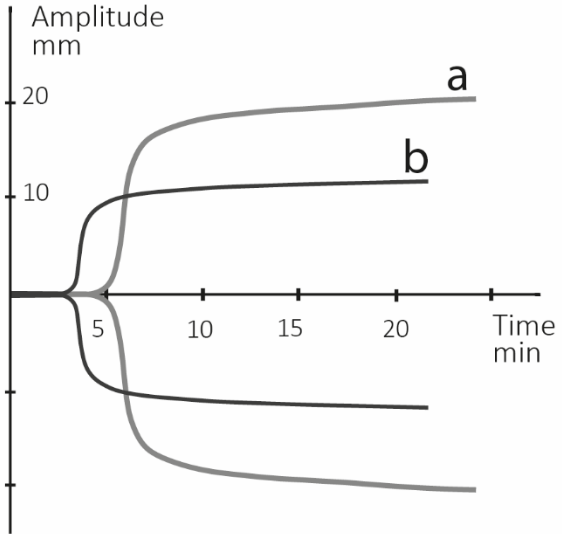

Figure S3. Characteristic thromboelastograms (TEGs) of a COVID-19 patient (**a**) and a healthy donor (**b**), illustrating the deceleration of clotting and an increase in the clot strength in clots from the COVID-19 plasma sample.
